# Revenant: a database of resurrected proteins

**DOI:** 10.1093/database/baaa031

**Published:** 2020-05-13

**Authors:** Matias Sebastian Carletti, Alexander Miguel Monzon, Emilio Garcia-Rios, Guillermo Benitez, Layla Hirsh, Maria Silvina Fornasari, Gustavo Parisi

**Affiliations:** 1 Departamento de Ciencia y Tecnología, CONICET, Universidad Nacional de Quilmes, Roque Saenz Peña 182, Bernal, B1876BXD, Buenos Aires, Argentina; 2 Department of Biomedical Sciences, University of Padova, Viale G. Colombo 3, Padova, I-35131, Padova, Italy; 3 Departamento de Ingeniería, Pontificia Universidad Católica del Perú, Lima, Perú

## Abstract

Revenant is a database of resurrected proteins coming from extinct organisms. Currently, it contains a manually curated collection of 84 resurrected proteins derived from bibliographic data. Each protein is extensively annotated, including structural, biochemical and biophysical information. Revenant contains a browse capability designed as a timeline from where the different proteins can be accessed. The oldest Revenant entries are between 4200 and 3500 million years ago, while the younger entries are between 8.8 and 6.3 million years ago. These proteins have been resurrected using computational tools called ancestral sequence reconstruction techniques combined with wet-laboratory synthesis and expression. Resurrected proteins are commonly used, with a noticeable increase during the past years, to explore and test different evolutionary hypotheses such as protein stability, to explore the origin of new functions, to get biochemical insights into past metabolisms and to explore specificity and promiscuous behaviour of ancient proteins.

## Introduction

**Figure 1:**
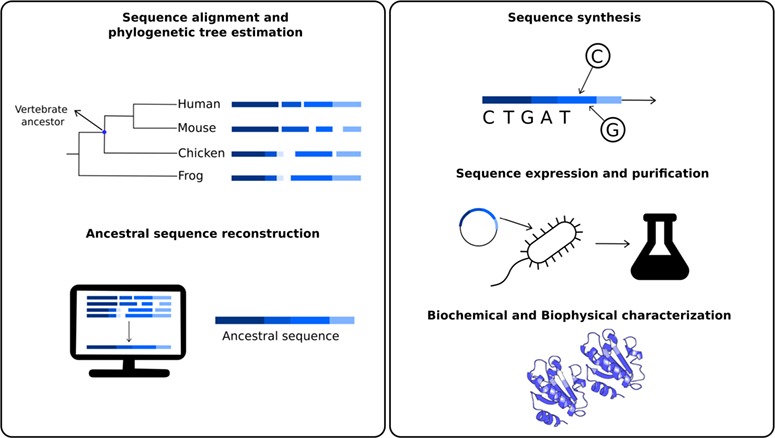
Schematic representation of the different steps to obtain resurrected proteins. The first step involves sequence similarity searches of a given protein to obtain a set of homologous sequences, involving the ancestral nodes to be studied. For example, one could be interested in studying biochemical properties of the studied protein in the last common ancestor for all vertebrates. Using these sequences, it is possible to estimate a phylogenetic tree to define the ancestral node to be reconstructed. In the second step, ancestral sequence reconstruction techniques are applied to estimate most probable sequences in the studied node. The third step involves the ancestral sequence synthesis. This sequence is then inserted into a vector, cloned, expressed and purified (fourth step). The fifth and final step involves a series of biochemical and biophysical characterization.

As a time machine, a combination of *in silico* and wet laboratory approaches allow the prediction of most probable sequences of proteins coming from organisms that lived millions of years ago (1). These predicted and synthesized sequences coming from extinct organisms are called resurrected proteins. Protein resurrection consists mainly in five steps (Figure 1) (2, 3). In the first one, a set of extant sequences, homologous to the ancestral protein to be studied, are aligned and used to estimate a phylogenetic tree. Phylogenetic trees are used to infer evolutionary relationships between ancestral and extant organisms. In a tree, extant organisms are represented by the terminal nodes or tips of the tree, (Figure 1), while the ancestral organisms are represented by internal nodes. As an example, Figure 1 indicated the node representing the extinct organism corresponding to the last common ancestor from all extant vertebrates, or at least for those present in the set of homologous sequences considered. Using these evolutionary relationships, in the second step, it is possible to infer the most probable sequence for each of the ancestral states using the so called ancestral sequence reconstruction (ASR) techniques. These methods comprise a set of computational tools using different algorithms such as maximum parsimony (4), maximum likelihood (5–7) as well as Bayesian methods (8). In the third step, the predicted ancestral sequences could be synthesized using molecular biology techniques. If the ancestral reconstruction involves recent ancestors, site-directed mutagenesis using an extant gene can be used to obtain the ancestral sequence (9). However, in those cases where remote proteins are resurrected, gene synthesis (10) or gene fragments assembly are required to obtain the ancestral gene. Once synthesized, the gene is cloned, expressed and purified (fourth step). Then, the protein could be further experimentally characterized and studied as any other present day protein (fifth step). Most of these experiments consist in different biochemical and biophysical characterizations and also structural determination using X-ray crystallography or nuclear magnetic resonance.

**Figure 2:**
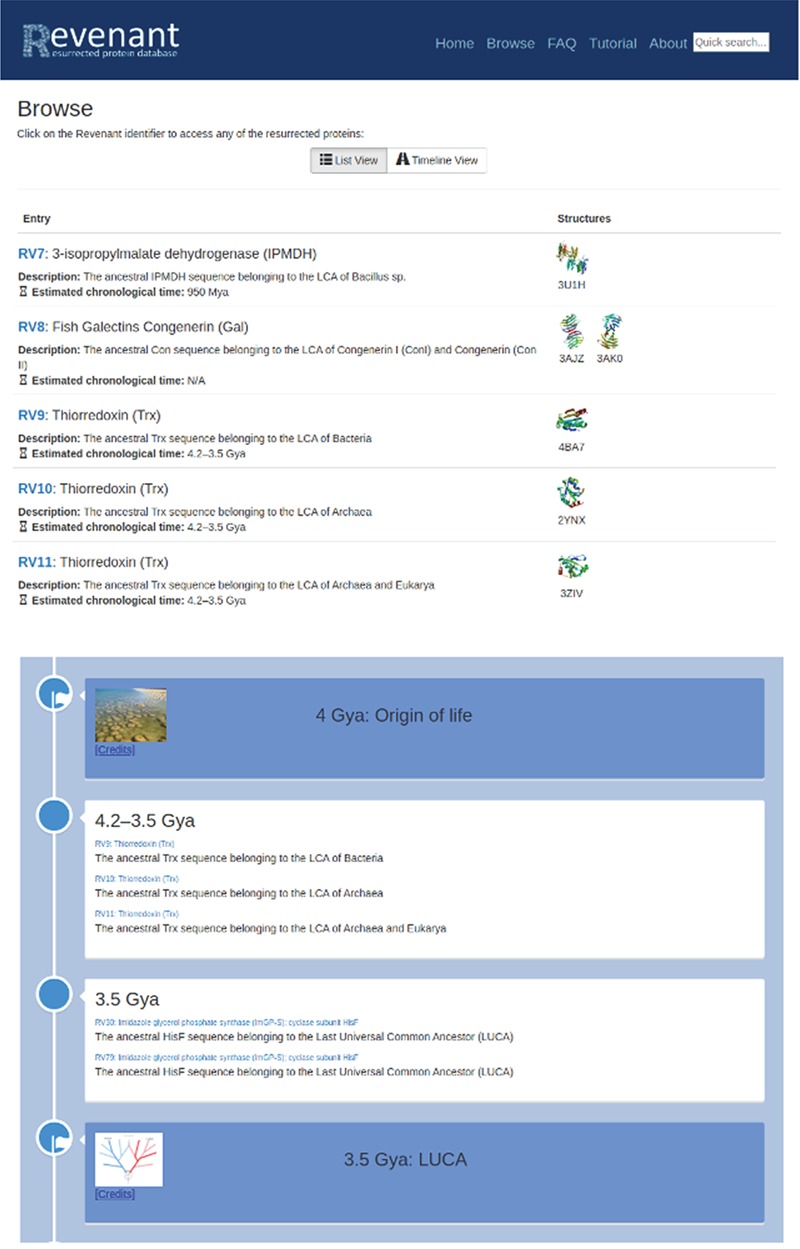
Two different browsing capabilities are available in Revenant. In the first one (top panel) proteins are listed sequentially using their RV codes. In the second browser (bottom panel) we display the Revenant proteins in an Earth’s timeline showing important biological events since the origin of life.

**Figure 3:**
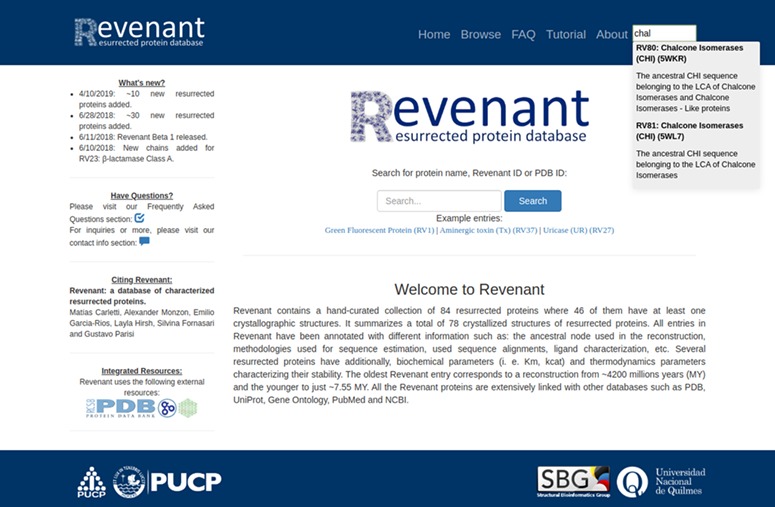
Screenshot of Revenant web server showing the home page and search utilities.

**Figure 4:**
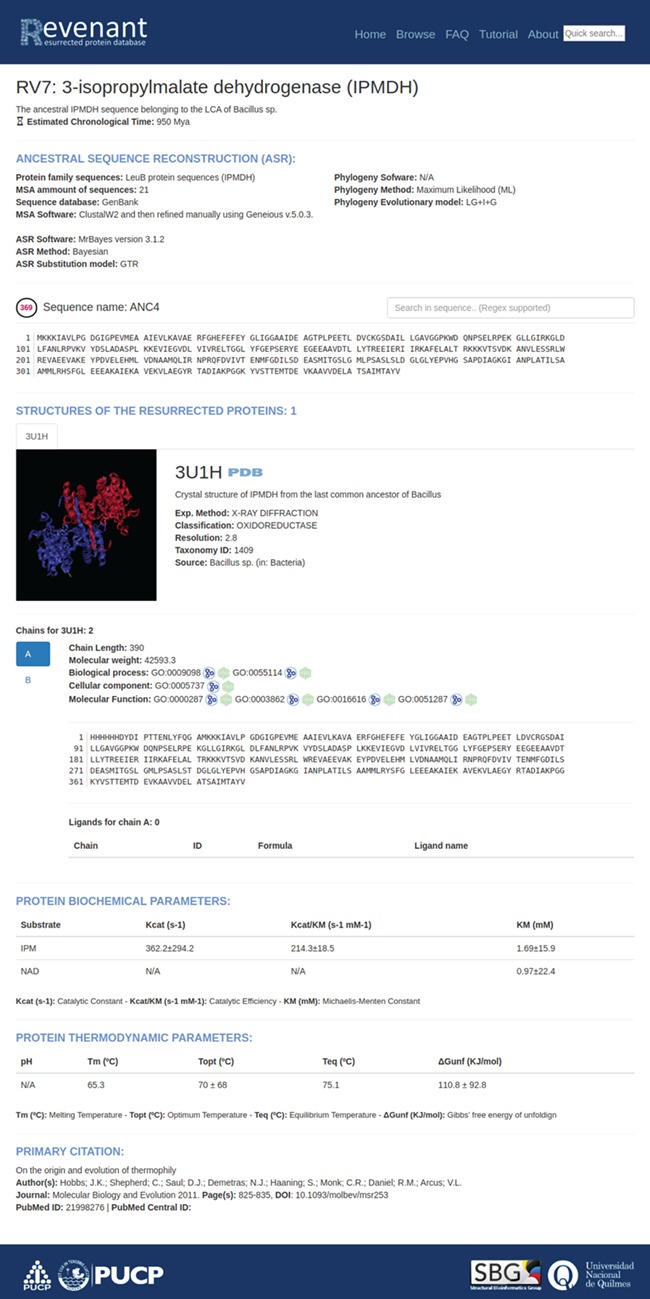
Main entry page. Each entry starts with a title followed by a brief explanation of the biological relevance of the resurrected protein. Additionally, each entry has fields regarding ancestral sequence reconstruction, information about their structures, biochemical and biophysical parameters and, finally, the primary citation.

The reliability of protein resurrection relies on the inference protocol used. Ambiguous estimation, dependence on tree topologies, approximations in evolutionary models and the difficulty to model indels are among the most common problems detected in resurrection (1, 2, 11–13). However, better results are obtained considering and minimizing those caveats. Furthermore, the creation of experimentally based phylogenies has contributed with a controlled system for ASR algorithms benchmarking (14).

Besides their caveats, ASR and protein resurrection are powerful strategies for testing different biological hypotheses. For example, phenotypic adaptations in dim-vision in vertebrates were recently elucidated using ancestral reconstruction and biochemical experimentation (15). Dim-vision in vertebrates is mediated with a family of proteins called rhodopsins. Slight variations in rhodopsin sequences during evolution confer the molecular basis of the spectral tuning observed in different organisms adapted to their environments (16). The authors found that 15 replacements (~3% of the average length of the rhodopsins) are essential to understand the functional adaptation. Moreover, some of these replacements occurred multiple times in vertebrate evolution strongly suggesting the existence of positive selection. Using positive selection analysis over a set of homologous proteins is a commonly used procedure to detect important positions associated with functional adaptations (17). However, this evolutionary pattern was not detected using rhodopsins evolutionary analysis, showing the importance of the use of ASR and biochemical characterization to unveil the evolution of dim-vision in vertebrates. In a similar way, the used of ASR and resurrection techniques has had a key role to address different biological questions, such as specificity and biological activity (18), stability (19), promiscuity (20), study of alternative evolutionary histories (21), epistasis (22), evolutionary analysis of visual pigments (23), rational engineering (24) and emergence of new active sites (25), influence of evolutionary trajectories (26) and effect of duplication in functional divergence (27) just to mention a few of a large list of examples. Interestingly, as phylogenies could be calibrated with fossil records, resurrected proteins could recreate most probable states of proteins spanning very different geological times. The most challenging resurrections are about the very beginning of life on Earth (~4000 millions of years (28)).

In this work we present Revenant, the first database of resurrected proteins. It contains a manually curated collection of resurrected proteins which have been biochemically, biophysically and/or structurally characterized. Revenant proteins span several millions of years. The oldest entry corresponds to a reconstruction age between 4200 and 3500 million years ago which corresponds to the thioredoxin protein (28) (RV9, RV10 and RV11) and the younger entries between 8.8 and 6.3 million years ago corresponding to uricase (29) (RV74). Revenant proteins could display unique ancestral features. As the explained above example with the rhodopsins, experimental assays on resurrected proteins could reveal their structural arrangements, conformational diversity and dynamisms, differential stability and ligand binding affinities. These piece of evidence, along with the use of molecular phylogenies, could represent extremely useful information to test hypotheses about the origin of promiscuity, conformational epistasis, structural divergence and functional diversification grounded on a large-scale analysis. Also, the availability of a curated database as Revenant could offer a resource for evaluating the impact of evolutionary trajectories (22) on broadly used bioinformatic methods as homology modelling (30) as well as to test mechanistic evolutionary models of proteins (31, 32).

## Database fields and contents

Each resurrected protein in Revenant represents the most probable sequence in a given node for a given phylogenetic analysis. Likewise, Revenant contains 84 entries (i.e. RV1-RV84) where 45 of them have at least one known crystallographic structure. Considering different structures of the same protein, Revenant contains a total of 78 crystallized structures of resurrected proteins. Using bibliographic information and manual curation, all entries have been annotated with different information such as the ancestral node used in the reconstruction, its estimated age, ASR methodologies used for sequence estimation, sequences and softwares used for the multiple alignments and phylogenetic estimation, structure availability and their ligand characterization and primary citation. Additionally, several entries have biochemical (i. e. Km, kcat) and biophysical parameters (i.e. melting temperature, ΔGunfolding). Furthermore, all the Revenant proteins are extensively linked with other databases such as PDB (33), UniProt (34), Gene Ontology (35) and PubMed.

## Database access and user interface

Resurrected proteins in Revenant can be easily found searching by protein family name and/or PDB code. The browser contains two modes for protein search, one displays all Revenant entries as a list and the other shows a geological timeline indicating the approximate age of each Revenant entry (Figure 2).

Using the search or browse capabilities it is possible to access all Revenant entries. They are displayed along with a short description about the resurrected protein and, when it is available, the approximate age of the ancestral node (Figure 3). Further information is displayed in four different sections (Figure 4) for each entry: `ancestral sequence reconstruction’ contains all the information related to the ASR approach used for a given reconstruction. It also shows the reconstructed sequence and its name. The `structures of the resurrected proteins’ section summarizes the information about available structures, ligands, chains and biological function. The third section contains information about protein biochemical parameters like k_cat_ and affinity constants (such as K_M_) for given ligands as well as thermodynamic parameters (such as T_opt_, T_m_, and ΔGunfolding). Actually, ~23% of the entries in Revenant contain physicochemical information. The fourth and last section shows information about primary citation where the protein was resurrected. Revenant website also contains Frequently Asked Questions (FAQ) and tutorial sections to allow non-expert users to easily explore the database.

## Implementation

Revenant database was designed with microservices architecture. Two main elements of the system are the presentation and data components. The presentation elements exchange data using a RESTful API and the JavaScript Object Notation. The Java programming language and Spring framework leverage the data component implementation. MySQL is used for data storage and the ReactJS framework is used for presentation. Revenant offers users both graphical web interface access and RESTful web services from http://revenant.inf.pucp.edu.pe/.

## Conclusions

Revenant database offers a well-curated, updated and annotated collection of resurrected proteins. We think that Revenant can be used to explore the fascinating world of the increasing examples of resurrected proteins and their use to illuminate interesting biological and evolutionary questions (36). Furthermore, our database of ancient proteins could also be a source of sequence, structure, conformational diversity and biochemical data to test further biological hypothesis and to develop new tools related with structural bioinformatics, 3D protein modelling and protein evolution.

## Notes

**Database URL:** http://revenant.inf.pucp.edu.pe/
